# Explainable Machine Learning Approaches Predict Frailty and Adverse Outcomes in Older Adults: Development and Validation with Two Longitudinal Cohorts

**DOI:** 10.3390/jcm15051812

**Published:** 2026-02-27

**Authors:** Aixuan He, Jiang Zhang, Xiuying Hu

**Affiliations:** 1 Innovation Center of Nursing Research and Nursing Key Laboratory of Sichuan Province, West China Hospital, Sichuan University, Chengdu 610065, China; heaixuan1@stu.scu.edu.cn; 2 West China School of Nursing, Sichuan University, Chengdu 610065, China; 3 College of Electrical Engineering, Sichuan University, Chengdu 610065, China

**Keywords:** older adult, frailty, adverse outcomes, machine learning, longitudinal studies

## Abstract

**Objectives**: Early and accurate identification of frailty is essential for preventing adverse outcomes in older adults. However, existing frailty prediction models often lack reliability, interpretability, and generalizability. **Methods**: Participants aged 60 years and older between 2011 and 2015 (n = 3419) from the CHARLS were used to develop models, and participants from the CLHLS-HF between 2014 and 2018 (n = 1017) were used for external validation. The frailty was assessed 4 years after baseline in both cohorts by Fried’s Frailty Phenotype (FFP). Six machine learning models were applied to develop prediction models. The SHapley Additive exPlanations (SHAP) method was utilized to explain the final model. Clinical outcomes were evaluated between participants predicted as frail and non-frail by the final model. **Results**: The XGBoost (AUC = 0.934, 95% CI: 0.921–0.948; F1 = 0.712, 95% CI: 0.686–0.736 in internal validation; AUC = 0.792, 95% CI: 0.750–0.830; F1 = 0.702, 95% CI: 0.652–0.753 in external validation) performed best among six models. Key predictors included lifestyle factors (e.g., instrumental daily living activities, BMI, and self-rated health) and psychological traits (e.g., depression). Participants predicted as frail had significantly elevated risks of falls (OR = 2.11), hospitalization (OR = 1.75), and disability (OR = 1.42). **Conclusions:** The proposed model provided a robust and interpretable digital tool for predicting frailty among older adults and associated adverse outcomes.

## 1. Introduction

Frailty is an age-related clinical syndrome characterized by diminished physiological reserve and increased vulnerability to stressors and adverse health outcomes [1,2,3]. A systematic review spanning 62 countries estimated the global prevalence of frailty among individuals aged 50 years and older to range from 7% to 24% [4], with rates expected to rise due to the global population aging. Frailty has been consistently associated with a range of adverse health outcomes, including increased risk of falls (hazard ratio (HR) = 1.31), hip fracture (HR = 1.64), hospitalization (HR = 1.36), disability (HR = 3.13), dementia (HR = 1.52), and mortality (HR = 2.14) [5]. These associations underscore the critical need for early and accurate prediction of frailty among older adults to support timely intervention and prevent downstream complications.

Traditional frailty assessment tools—such as FFP [2], the Frailty Index (FI) [6], the Clinical Frailty Scale [7], and the Vulnerable Elders Survey [8]—are primarily designed for diagnosing or screening frail individuals rather than predicting future frailty onset. These instruments are static and rely on periodic physical examinations and clinical assessments, which limit their utility for detection and proactive management. A significant gap exists for a tool that is capable of leveraging longitudinal data to estimate an individual’s future status of frailty. Machine learning (ML)-based models, increasingly developed from electronic health records and population-based databases, offer a dynamic and individualized approach to frailty prediction [9,10,11,12,13,14]. ML algorithms can continuously update predictions using longitudinal health data, enabling timely risk identification and personalized preventive strategies. Additionally, ML models are capable of iterative self-improvement through deployment, enhancing predictive accuracy and robustness over time. Compared with static assessment tools, ML approaches provide superior adaptability, scalability, and real-time processing capabilities—making them particularly well-suited for population-level frailty screening and public health interventions. 

Although several studies have employed ML approaches to develop frailty prediction models [9,10,13], existing efforts present several limitations. First, interpretability remains a key challenge in most existing ML-based models. Many ML models function as “black boxes,” offering limited transparency into the decision-making process and reducing their clinical trustworthiness and acceptance. Second, external validation is often absent, which restricts the generalizability and real-world applicability of these models [15]. Third, few studies systematically evaluate the prognostic utility of predicted frailty status to further impact clinical decision-making and care management for older adults. These limitations highlight the need for frailty prediction models that are not only accurate but also interpretable, transparent, and externally validated to support their integration into clinical and public health practice.

To address these limitations, this study aimed to: (1) develop interpretable ML models to predict frailty within 4 years among older adults, incorporating SHAP to enhance model transparency; (2) conduct both internal and external validation of model performance using two large, population-based longitudinal cohorts; and (3) evaluate the prognostic utility of model-predicted frailty by assessing its associations with adverse outcomes, including falls, hospitalizations, and disability.

## 2. Materials and Methods

### 2.1. Data Source and Study Participants

The frailty prediction models were developed using data from the China Health and Retirement Longitudinal Study (CHARLS) and externally validated using data from the Chinese Longitudinal Healthy Longevity and Happy Family Study (CLHLS-HF). CHARLS is a nationally representative, prospective cohort designed to collect health, demographic, and economic data from middle-aged and older adults in China [16]. The baseline survey was conducted in 2011–2012 using a probability-proportional-to-size sampling method to construct a nationally representative sample, followed by subsequent waves in 2013, 2015, 2018, and 2020. The survey first randomly selected 150 county-level units across the country as primary sampling units, and further randomly selected three villages or neighborhoods within each chosen county or district, covering a total of 450 communities. All members and their spouses from each selected household were invited to participate in the survey. This design ensured that the baseline sample could be effectively generalized to the middle-aged and elderly population in China. The study enrolled 17,708 individuals aged 45 years and older and collected a wide range of data, including self-reported health status, physical examinations, biomarkers, and socioeconomic indicators. CLHLS-HF is a nationwide longitudinal study focusing on individuals aged 65 years and older [17]. The study began in 1998, with follow-up surveys conducted in 2000, 2002, 2005, 2008–2009, 2011–2012, 2014, and 2017–2018, enrolling 15,874 participants. It collected comprehensive information on demographic characteristics, lifestyle behaviors, physical and cognitive function, and psychological well-being. Both CHARLS and CLHLS-HF received ethical approval from the Peking University Biomedical Ethics Committee (CHARLS approval number: IRB00001052-11015; CLHLS-HF approval number: IRB00001052-13074). Written informed consent was obtained from all participants at enrollment.

Baseline data were derived from the 2011 wave of CHARLS and the 2014 wave of CLHLS-HF. Frailty status was determined using follow-up data collected four years later in each cohort. Participants were excluded if they met any of the following criteria: (1) age < 60 years at baseline; (2) classified as frail at baseline; (3) missing frailty assessment data; or (4) lost to follow-up or deceased at the time of outcome assessment.

### 2.2. Assessment of Frailty

The primary outcome of this study was frailty, assessed using the modified FFP criteria. The original FFP criteria included five domains: shrinking, weakness, slowness, exhaustion, and inactivity. Shrinking was defined as a body mass index (BMI) of <18.5 kg/m^2^ [18,19]. Weakness was assessed by the inability to lift a 5 kg bag [20]. Slowness, exhaustion, and inactivity were derived from validated self-reported health questionnaire items available in CHARLS and CLHLS-HF due to data availability constraints in these publicly available longitudinal datasets [21,22]. Weakness was assessed using self-reported difficulty lifting weights rather than grip strength measurements, and slowness was assessed using self-reported mobility limitations rather than timed walk tests. Each domain was scored as one if present, and participants with a total score of ≥3 were classified as frail [23]. Participants with more than two missing values were excluded. Those with two missing values were retained if they still met the frailty threshold (i.e., a total score of 3). Detailed operational definitions for FFP are provided in Supplementary Table S1.

### 2.3. Potential Predictors

Candidate predictors of frailty were selected based on clinical relevance, prior literature evidence, and availability across both the CHARLS and CLHLS-HF datasets. A total of 39 variables were identified for inclusion in the initial screening process for ML model development. These variables encompassed sociodemographic characteristics (e.g., age, gender, education level), health and lifestyle factors (e.g., BMI, waist circumference, pulse, diastolic blood pressure, smoking status, alcohol consumption), psychosocial and mental health indicators (e.g., depressive symptoms, cognitive function, and sleep problems), and laboratory measures (e.g., white blood cell count, hemoglobin, hematocrit). Depression was evaluated using the 10-item Center for Epidemiological Studies Depression Scale (CESD-10), with a score of ≥10 indicating clinically relevant depressive symptoms. Cognitive function was measured by the Mini-Mental State Examination (MMSE), with total scores ranging from 0 to 30, where higher scores indicate better performance [24,25]. Detailed variable definitions and coding schemes for each dataset are provided in Supplementary Table S2.

### 2.4. Model Development and Validation

The dataset was randomly split into a training and testing set at a 7:3 ratio. Furthermore, 10-fold cross-validation was employed within the training set. The dataset was randomly partitioned into 10 subsets. In each interaction, one subset was used as a validation fold, while the remaining nine were used as training folds. The process was repeated 10 times, and the average performance across all folds was calculated.

In data preprocessing, standardization, imputation, and class imbalance processing were performed. Before model training, all input variables were standardized to a mean of 0 and a standard deviation of 1 to ensure consistent scaling and prevent features with larger magnitudes from disproportionately influencing model learning. Missing data were handled using the missForest, a widely recognized imputation method based on the random forest (RF) algorithm [26]. The missForest method leverages the observed data structure to predict missing values through iterative RF modeling, preserving non-linear interactions between variables. This approach is capable of handling both continuous and categorical variables and reduces bias compared to the single imputation method [27]. Furthermore, the Synthetic Minority Oversampling Technique (SMOTE) was applied to address the class imbalance between frail and non-frail participants [28]. SMOTE generates synthetic samples for the minority class based on the K-nearest neighbors (K-NN) algorithm, thereby improving model performance in imbalanced classification tasks. All ML models were subsequently trained on the training set and evaluated on the test set. To prevent data leakage and ensure unbiased performance estimation, data preprocessing was performed exclusively on training folds within the training set.

In feature selection, feature selection was performed using the least absolute shrinkage and selection operator (Lasso) regression, a penalized regression method that enhances model interpretability by shrinking less important coefficients to zero [29]. The Lasso algorithm applies an L1 penalty to the regression model, effectively performing variable selection by retaining only the most predictive features while excluding redundant or irrelevant ones. This approach was employed primarily for dimensionality reduction and to enhance model interpretability. Lasso regression was not identified as an optimal feature selector for subsequent non-linear ML algorithms, which possess inherent capabilities to handle high-dimensional data and capture complex feature interactions. Features with non-zero coefficients after Lasso regularization were retained for subsequent model development. 

The model development process consisted of two main phases: derivation and internal validation. Six ML algorithms—K-NN, support vector machine (SVM), RF, Gradient Boosting Machine (GBM), XGBoost, and CatBoost—were used to construct frailty prediction models. In addition, traditional LR was implemented as a baseline comparator. All ML models were trained using a nested cross-validation framework to prevent information leakage and optimistic bias. Within each outer fold, an inner cross-validation loop was performed on the outer training folds for hyperparameter optimization via grid search. The Lasso regression parameter was treated as a hyperparameter through grid search. In the end, ML models with the optimal hyperparameter combination were trained on the 70% training set and evaluated on the 30% testing set. External validation was subsequently performed using the CLHLS-HF dataset to assess the generalizability and performance of the final models in an independent population.

In model performance evaluation, evaluation metrics included the AUC, precision, recall, F1-score, and accuracy. To account for sampling variability and support statistical comparison, 95% confidence intervals (CIs) for indicators were calculated through 1000 bootstrap resamples. The optimal model was selected based on the highest values of AUC and F1-score, with AUC prioritized as the decisive criterion in cases of discordant results [30]. A model was considered to exhibit good discrimination ability if it achieved an AUC > 0.707 [31].

### 2.5. Interpretation Analysis

To enhance the transparency and address the inherent black-box nature of ML models, this study employed the SHAP for post hoc interpretability. SHAP assigns each feature a value representing its contribution to the model’s predictions, thereby offering insights into how individual predictors influence the outcome. For global interpretability, SHAP values were averaged across all samples to quantify the overall importance of each feature in model decision-making. For local interpretability, SHAP values were computed at the individual level, enabling case-specific explanations and validating the consistency of predictions [32]. Both global and individual SHAP analyses were conducted to provide a comprehensive understanding of model behavior and support the clinical credibility of the predictive outputs.

### 2.6. Statistical Analysis

All statistical analyses were performed using Python (version 3.12.4) and IBM SPSS (version 27.0). Continuous variables were summarized as mean ± standard deviation (SD) or median with interquartile range (IQR) for non-normally distributed data, while categorical variables were reported as frequencies and percentages. Group comparisons were conducted using the Pearson χ^2^ test for categorical variables and the independent samples t-test or Mann-Whitney U test for continuous variables, as appropriate. Model performance was evaluated using AUC, precision, recall, F1-score, and accuracy. To assess the prognostic value of ML-predicted frailty status, a binary logistic regression was applied to examine associations with adverse outcomes. A three-model adjustment strategy was employed to evaluate the robustness of associations and address potential confounding. Model 1 (unadjusted) included only predicted frailty status as the exposure, model 2 adjusted for demographics (age and gender), and model 3 additionally adjusted for socioeconomic factors (education level and marital status). These covariates were selected based on established associations with adverse outcomes [33,34,35,36]. To prevent overadjustment, health-related covariates that overlap with ML predictors were excluded. A two-tailed *p*-value < 0.05 was considered statistically significant.

## 3. Results

### 3.1. Baseline Characteristics

**Figure 1:**
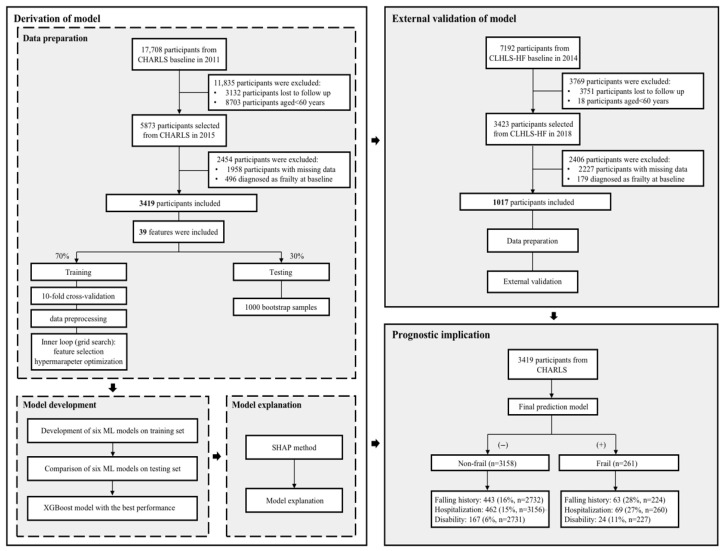
The flowchart of the study design.

3419 eligible participants were included from the CHARLS cohort. In the CLHLS-HF cohort, 1017 participants met the FFP criteria (Figure 1). 

In the model development cohort (CHARLS), 9.2% (n = 316) of participants were classified as frail. The median age was 65 years (IQR: 62–70), and 45.1% were female. In the external validation cohort (CLHLS-HF), 18.1% (n = 184) were classified as frail. The median age was 78 years (IQR: 71–86), with 39.7% female. Baseline characteristics of the two cohorts are compared in Table 1.

### 3.2. The Result of Predictor Selection and Model Development

**Figure 2:**
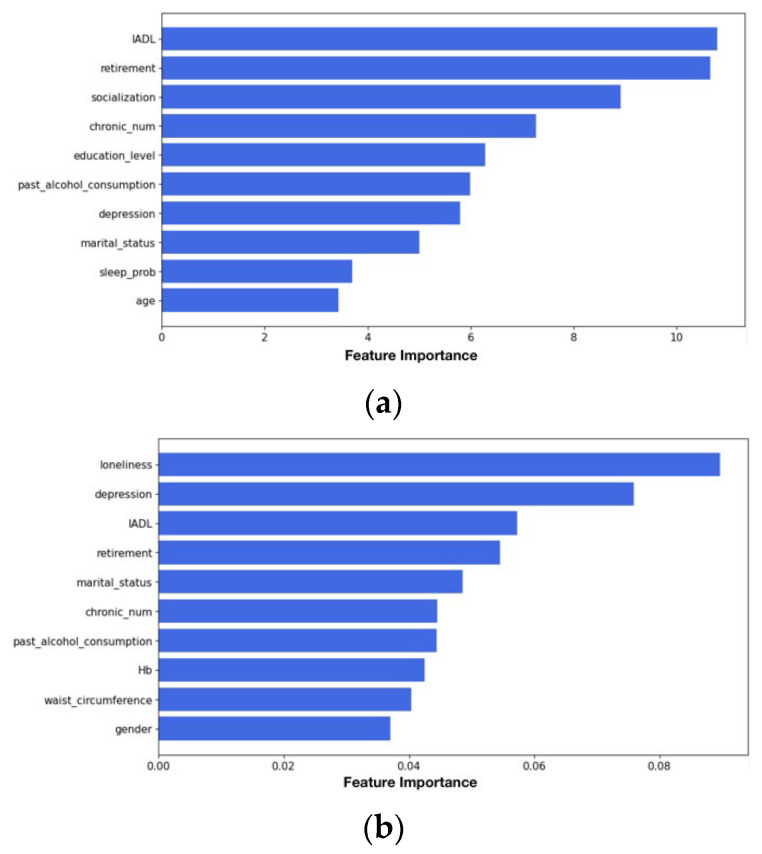
Bar plots of the relative importance of selected features in the CHARLS cohort based on (**a**) XGBoost, and (**b**) CatBoost.

Figure 2 presents bar plots of feature importance based on the XGBoost and CatBoost models. In the XGBoost algorithm, the top five predictors identified were instrumental activities of daily living (IADL), retirement, socialization, number of chronic conditions, and education level. The Lasso algorithm excluded current alcohol consumption. In the CatBoost algorithm, the top five predictors identified were loneliness, depression, IADL, retirement, and marital status. Triglycerides, glucose, and total cholesterol were excluded by the Lasso algorithm. 

### 3.3. Model Performance in Internal Validation

**Figure 3:**
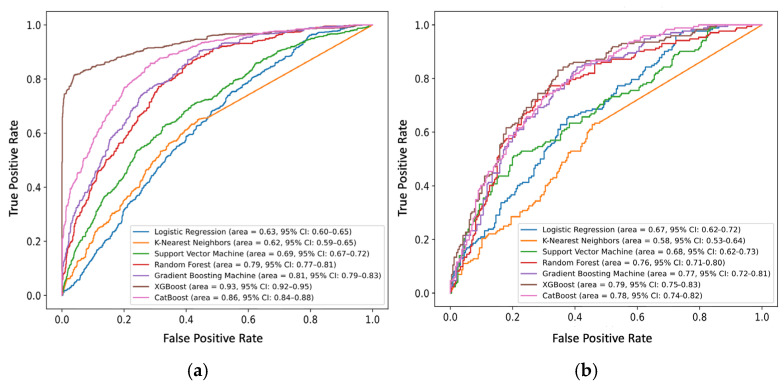
Model performance based on ROC curves in (**a**) CHALRS and (**b**) CLHLS-HF cohort.

Frailty prediction models were developed using six ML algorithms and LR. For FFP-based prediction, the XGBoost achieved the highest performance (AUC: 0.934, 95% CI: 0.921–0.948; F1: 0.712, 95% CI: 0.686–0.736), followed by CatBoost (AUC: 0.862, 95% CI: 0.844–0.879; F1: 0.760, 95% CI: 0.737–0.784) and GBM (AUC: 0.814, 95% CI: 0.793–0.834; F1: 0.712, 95% CI: 0.686–0.736). The KNN model showed the lowest performance (AUC: 0.621, 95% CI: 0.593–0.647; F1: 0.546, 95% CI: 0.512–0.576). RF, GBM, XGBoost, and CatBoost models demonstrated acceptable predictive accuracy (Figure 3a).

### 3.4. Model Performance in External Validation

In the external validation cohort, XGBoost achieved the highest discriminative performance (AUC: 0.792, 95% CI: 0.750–0.830; F1: 0.702, 95% CI: 0.652–0.753), followed by CatBoost (AUC: 0.781, 95% CI: 0.739–0.822; F1: 0.678, 95% CI: 0.624–0.843) and GBM (AUC: 0.768, 95% CI: 0.723–0.811; F1: 0.656, 95% CI: 0.596–0.711). These three models consistently outperformed others (Figure 3b). Based on AUC thresholds, the XGBoost, CatBoost, GBM, and RF models demonstrated acceptable discriminative performance for FFP-based frailty prediction. Model performance for all ML models is demonstrated in Supplementary Table S3. 

### 3.5. Clinical Interpretation

**Figure 4:**
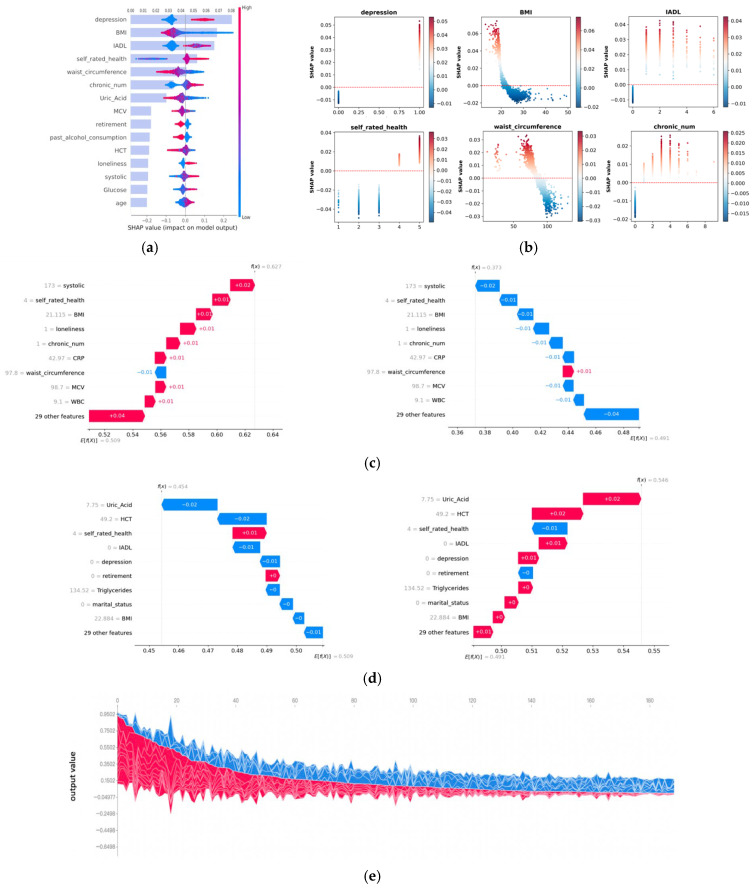
Results of SHAP analysis. (**a**,**b**) Global interpretability analysis with SHAP. (**a**) SHAP summary plot for all samples. The bar plot presents mean absolute SHAP values in descending order, and the scatter plot presents the distribution of SHAP values, with red indicating higher feature values and blue indicating lower feature values. (**b**) SHAP dependence plots for the top 6 features. The x- and y-axes represent the value of the variable and the SHAP value, respectively. (**c**–**e**) Local interpretability analysis with SHAP. Waterfall plots for a sample predicted as (**c**) frail and (**d**) non-frail. The left graphs represent the result towards the frailty class, and the right graphs represent the result towards the non-frailty class, with red indicating positive SHAP values and blue indicating negative SHAP values. (**e**) force plot for 200 samples, where red represents contributions pushing the prediction towards frailty, and blue represents contributions pushing towards non-frailty.

SHAP analysis was performed in the CHARLS cohort. The XGBoost model was selected for SHAP analysis due to its robust predictive performance in both internal and external validation. To explore the XGBoost models’ decision-making process, both global and individual interpretability approaches were conducted. For global interpretability, SHAP values were aggregated across all participants to estimate the average contribution of each feature to the XGBoost model (Figure 4a). The bar plot displays the mean absolute SHAP values, reflecting the overall importance of each predictor in frailty prediction. Depression, BMI, IADL, self-rated health, and waist circumference emerged as the most influential features. A corresponding SHAP scatter plot illustrates the distribution of feature contributions.

Figure 4b presents SHAP dependence plots for the top six features with the highest mean SHAP values. These plots illustrate the marginal contributions of individual features to the model’s output and reveal how specific values of each factor affect frailty status. For categorical variables, depression, IADL disability, a higher number of chronic conditions, and higher self-rated health were identified as positive contributions to frailty prediction. Among continuous variables, lower values of BMI and waist circumference are significant contributors, indicating that lower body mass is associated with an increased predicted probability of frailty.

Individual-level interpretation was conducted to examine the contribution of each feature to the predicted outcome for a single sample. In the SHAP waterfall plot, red bars represent positive contributions to frailty prediction, while blue bars indicate negative contributions. To illustrate the clinical application of local explanations, two representative cases were selected. A typical case predicted as frail (Figure 4c) and a typical case predicted as non-frail (Figure 4d). Figure 4c illustrates a single sample that was identified as frail. The left panel displays the cumulative impact of all features, resulting in a frail status with a probability of 0.627, while the right panel shows the corresponding low probability (0.373) for the non-frail class. Key features contributing positively to the prediction included systolic blood pressure, self-rated health, BMI, loneliness, and the number of chronic diseases.

Figure 4d illustrates an example of a non-frail prediction, with a frailty probability of 0.454 (left panel) and a non-frailty probability of 0.546 (right panel). In this case, the prediction was largely driven by contributions from uric-acid level, hematocrit level, depression, IADL, and retirement, among others. Additionally, Figure 4e presents a SHAP force plot for 200 participants. Each individual is represented along the *x*-axis, with the cumulative feature contributions shown on the *y*-axis. Red areas denote features contributing positively to frailty prediction, enabling a visual comparison of feature effects across samples.

### 3.6. Prognostic Analysis

To assess the prognostic implications of model-predicted frailty, outcomes predicted by the XGBoost model were further analyzed based on its superior performance. A total of 3419 participants from the CHARLS cohort were included to examine associations between predicted frailty status and adverse outcomes. Frailty status predicted by the XGBoost model was used as the exposure variable in a binary logistic regression model. Outcomes included history of falls, hospitalization, and disability (Table 2). Compared to individuals classified as non-frail, those identified as frail had significantly increased odds of falling (Odds Ratio (OR) = 2.11), hospitalization (OR = 1.75), and disability (OR = 1.42). 

## 4. Discussion

This study developed six ML models—K-NN, SVM, RF, GBM, XGBoost, and CatBoost—using the CHARLS cohort to predict frailty among older adults. External validation was conducted using the CLHLS-HF cohort. Among all models, XGBoost demonstrated the most consistent and superior predictive performance across both internal and external validation. The SHAP-based interpretability analysis revealed depression, BMI, IADL, self-rated health, and waist circumference as the key contributors to frailty prediction in the XGBoost model. Furthermore, frailty, as predicted by XGBoost, was significantly associated with adverse outcomes, including falls, hospitalization, and disability. 

The prevalence of frailty and model performance varied depending on the diagnostic criteria used. In the model development cohort (CHARLS), frailty prevalence was 9.2% based on FFP. These estimates aligned with previous epidemiological data from systematic reviews (12% for FFP) [4] and other CHARLS-based studies among older adults (8.1% for FFP [37]). In the external validation cohort (CLHLS-HF), frailty prevalence was 18.1% (FFP), which is comparable to prior CLHLS-HF studies. Notably, the wider variability in FFP-based prevalence was observed across CLHLS-HF reports (ranging from 26.3% [22] to 37.5% [38]).

Generally, all ML models outperformed traditional LR in terms of predictive performance. Among them, GBM, XGBoost, and CatBoost consistently showed superior discrimination across both cohorts and frailty definitions. Their performance advantage likely stems from their ensemble learning mechanisms, which aggregate predictions from multiple weak learners to improve generalizability and reduce variance. 

The XGBoost-based frailty prediction model demonstrated strong and consistent performance across multiple evaluation metrics. Direct comparison with prior ML frailty studies reveals that our XGBoost model (AUC: 0.934 internal, 0.792 external) compares favorably with recent published models. Zhang et al. achieved an AUC 0.75 for the RF model in predicting frailty among Chinese elderly by applying longitudinal data from the CLHLS-HF [39]. Wu et al. reported AUC 0.702 for RF predicting frailty trajectories in CLHLS-HF [14]. However, different study designs, study populations, and validation strategies should be considered. In addition to its predictive accuracy, the inclusion of 39 clinically relevant predictors, covering sociodemographic and health-related factors, improved the models’ interpretability. SHAP analysis confirmed that several key predictors, such as age, IADL, and BMI [40,41], have been previously associated with frailty in the literature [42,43,44,45], reinforcing the clinical validity of the model. Moreover, the model’s ability to identify individuals at elevated risk of adverse outcomes further supports its potential utility in early intervention strategies [46].

Several limitations should be considered when interpreting the findings. First, the models were trained and validated using cohorts composed exclusively of older Chinese adults, which may limit generalizability to other populations. Further studies should aim to include more diverse cohorts across geographic, ethnic, and healthcare system contexts. Second, a modified version of the FFP was applied due to constraints in publicly available datasets. The use of self-reported measures for exhaustion and physical activity introduces subjectivity and potential recall bias. As more comprehensive datasets become available, incorporating the original FFP criteria may improve comparability across studies. Future studies should validate our model against objectively measured frailty criteria and assess whether prediction accuracy improves with the incorporation of performance-based assessments. Third, several methodological limitations should be considered. Missing data were imputed using missForest, which was effective for handling complex missing-data patterns. The assumption it relies on may not fully capture the underlying data structures, potentially introducing bias. Alternative imputation methods, such as the more sophisticated Multiple Imputation by Chained Equations algorithm, can be adopted when sufficient computational resources are available. Although the pipeline was designed to avoid data leakage caused by the SMOTE algorithm, it may introduce the generation of noise samples. Furthermore, the absence of calibration analysis limits the ability to assess the reliability of predicted probabilities, as it prevents their use for absolute frailty prediction. However, the model remains valid for risk stratification through ranking individuals according to relative likelihood. Future research should incorporate a calibration assessment to ensure the clinical utility of predicted probabilities. Fourth, the current models rely on structured data. Incorporating multimodal information such as genomics, imaging, or sensor-derived data could enhance predictive accuracy and provide deeper insights into the underlying biology of frailty. Fifth, this study used a binary frailty prediction, excluding the pre-frailty state. Further research could adopt ordinal or continuous frailty scoring systems to better capture the frailty spectrum and support individualized risk stratification. Finally, for real-world implementation, integrating this prediction tool into Electronic Health Records (EHR) systems could enable automated patient screening and facilitate early intervention for individuals predicted as frail in clinical practice. Future studies should evaluate the feasibility, acceptability, and clinical effectiveness of EHR-integrated frailty prediction tools.

## 5. Conclusions

In conclusion, the XGBoost-based frailty prediction model demonstrated strong predictive performance across internal and external validation, demonstrating its reliability, interpretability, and generalizability. The model’s outputs were significantly associated with adverse outcomes, including falls, hospitalizations, and disability, underscoring its clinical relevance. This study provided a practical and explainable tool for early identification of frailty in older adults and guided the implementation of targeted preventive interventions in geriatric care.

## Figures and Tables

**Table 1:** Baseline characteristics across groups.

Characteristics	CHARLS					CLHLS-HF					*p* ^a^
	Total (n = 3419)	Non-Frail (n = 3103)	Frail (n = 316)	*p*	Effect Size ^c^ (95% CI)	Total (n = 1017)	Non-Frail (n = 833)	Frail (n = 184)	*p*	Effect Size (95% CI)	
Age (year, IQR)	65 (62–70)	65 (62–70)	66 (63–71)	0.025	−0.108 (−0.224, 0.007)	78 (71–86)	71 (77–84)	85 (78–92)	<0.001	0.552 (0.431, 0.673)	<0.001
Gender				<0.001	0.058 (0.024, 0.092)				<0.001	0.092 (0.049, 0.135)	0.002
Female	1543 (45.1%)	1367 (44.1%)	176 (55.7%)			404 (39.7%)	304 (36.5%)	100 (54.3%)			
Male	1876 (54.9%)	1736 (55.9%)	140 (44.3%)			613 (60.3%)	529 (63.5%)	84 (45.7%)			
Educational level				0.069	0.028 (0.002, 0.056)				<0.001	0.131 (0.088, 0.174)	<0.001
Illiterate	1006 (29.4%)	897 (29.0%)	107 (33.9%)			463 (45.5%)	341 (40.9%)	122 (66.3%)			
Educated	2413 (70.6%)	2204 (71.0%)	209 (66.1%)			554 (54.5%)	492 (59.1%)	62 (33.7%)			
Marital status				0.874	0.003 (0.001, 0.006)				<0.001	0.126 (0.083, 0.169)	<0.001
Married	2693 (78.8%)	2443 (78.7%)	250 (79.1%)			603 (59.3%)	532 (63.9%)	71 (38.6%)			
Other	726 (21.2%)	660 (21.3%)	66 (20.9%)			414 (40.7%)	301 (36.1%)	113 (61.4%)			
BMI ^b^ (kg/m^2^, IQR)	23.1 (21.1–25.0)	23.2 (21.2–25.1)	21.9 (19.7–24.2)	<0.001	0.339 (0.223, 0.455)	22.8 (22.8–24.6)	22.5 (20.4–24.8)	21.1 (19.3–23.7)	<0.001	0.412 (0.291, 0.533)	<0.001
Waist circumference (cm, IQR)	85.0 (79.2–91.1)	85.1 (79.8–91.4)	82.1 (75.9–88.2)	<0.001	0.313 (0.197, 0.429)	82.0 (76.0–89.0)	83.0 (77.0–89.0)	79.0 (74.0–88.0)	<0.001	0.198 (0.077, 0.319)	<0.001
Pulse (bpm, IQR)	71.2 (66.0–76.0)	71.1 (66.0–76.0)	71.8 (66.6–78.0)	0.021	−0.170 (−0.285, −0.054)	75.0 (68.0–80.0)	75.0 (68.0–80.0)	76.5 (70.0–81.0)	0.042	−0.124 (−0.245, −0.003)	<0.001
Diastolic blood pressure (mmHg, IQR)	74.3 (68.5–80.5)	74.4 (68.5–80.5)	73.5 (67.5–79.5)	0.240	0.054 (−0.062, 0.170)	80.0 (73.0–87.5)	80.0 (74.5–87.5)	80.0 (72.5–85.4)	0.513	−0.029 (−0.150, 0.092)	<0.001
Systolic blood pressure (mmHg, IQR)	132.0 (120.5–144.5)	132.0 (120.5–144.0)	131.1 (119.6–144.9)	0.899	−0.029 (−0.145, 0.087)	140.2 (128.0–156.0)	140.0 (129.0–157.0)	141.1 (126.4–155.0)	0.979	0.001 (−0.120, 0.122)	<0.001

^a^ denotes the *p* of the overall table for CHARLS and CLHLS-HF; ^b^ BMI: body mass index; ^c^ Cohen’s d for continuous variables, and Cramér’s V for categorical variables. Interpreted according to Cohen’s criteria: negligible (<0.1), small (0.1–0.3), medium (0.3–0.5), large (≥0.5).

**Table 2:** Logistic regression for predictive validity of the ML model against adverse outcomes.

		Model 1 ^a^	Model 2 ^b^	Model 3 ^c^
Outcome	Total n	OR (95% CI)	OR (95% CI)	OR (95% CI)
Falling history				
Non-frail	443	1.0	1.0	1.0
Frail	63	2.11 (1.27–3.50)	2.03 (1.21–3.26)	1.90 (1.15–3.14)
Hospitalization				
Non-frail	462	1.0	1.0	1.0
Frail	69	1.75 (1.21–2.53)	1.65 (1.14–2.39)	1.62 (1.15–2.24)
Disability				
Non-frail	167	1.0	1.0	1.0
Frail	24	1.42 (1.23–1.64)	1.41 (1.21–1.63)	1.46 (1.02–2.11)

^a^ Model 1 denoted the model without adjusting for any covariates; ^b^ Model 2 adjusted for age and gender; ^c^ Model 3 adjusted for age, gender, education level, and marital status.

## Data Availability

Publicly available datasets were analyzed in this study. These data can be found at http://opendata.pku.edu.cn/, accessed on 19 August 2024.

## Supplementary Materials

The following supporting information can be downloaded at: https://www.mdpi.com/article/10.3390/jcm15051812/s1, Table S1: The criteria of the modified Fried’s Frailty Phenotype; Table S2: Measurement of variables; Table S3: Model performance in the internal and external validation cohort; Table S4: TRIPOD checklist.
