# Supplementary material for: Explainable Machine Learning Approaches Predict Frailty and Adverse Outcomes in Older Adults: Development and Validation with Two Longitudinal Cohorts
Source: J Clin Med. 2026 Feb 27;15(5):1812. doi: 10.3390/jcm15051812 (PMC12986504; doi:10.3390/jcm15051812)
Supplement: Supplementary file 1 [file jcm-15-01812-s001.zip › jcm-4112721-supplementary.pdf]

Supplementary Materials

Supplementary Table S1. The criteria of the modified Fried's Frailty Phenotype

Supplementary Table S2. Measurement of variables

Supplementary Table S3. Model performance in the internal and external validation cohort

Supplementary Table S4. TRIPOD checklist

Supplementary Table S1

The criteria of the modified Fried's Frailty Phenotype.

**Item**	**Definition in CHARLS**	**Definition in CLHLS-HF**
Shrinking	Body mass index < 18.5 kg/m^2	
Slowness	Have difficulty walking 100m or climbing several flights of stairs without resting	Failed to walk for 1 km
Weakness	Failed to lift a bag weighing 5 kg	
Exhaustion	Either question:<br>- *I felt everything I did was an effort during last week*<br>- *I could not get going during last week*<br>with responses of *"most or all of the time"* or *"occasionally or a moderate amount of the time"*.	Either question:<br>- *I felt old and useless*<br>- *I felt everything I did was an effort*<br>with responses of *"always," "often," or "sometimes."*
Inactivity	If the participant did not undertake physical activity or walk at least 10 min at a time during a usual week	If the participant did the following activities 1 time per week or less: housework, outside activity, gardening, keeping a pet, livestock breeding, playing cards or mah-jongg, and social activity

Supplementary Table S2
Measurement of variables.

Predictors		Values	Code in CHARLS	Code in CLHLS-HF
**Sociodemographic characteristics**				
1	Age	year	r1agey	trueage
2	Gender	male=0, female=1	ragender	a1
3	Educational level	illiterate=0, educated=1	r1educ_c	f1
4	Marital status	single/divorced/widowed =0, married=1	r1mstat	f41
5	Retirement	No=0, Yes=1	r1retemp	f21
**Health and lifestyle related factors**				
6	Body mass index	kg/m2	r1mbmi	g101 & g1021
7	Waist circumference	cm	r1mwaist	g102c
8	Pulse	beats/minute	r1pulse	g7
9	Diastolic blood pressure	mmHg	r1diasto	g512 & g522
10	Systolic blood pressure	mmHg	r1systo	g511 & g521
11	Vision problem	Yes = 1, No = 0	DA005_3_	g1
12	Hearing problem	Yes = 1, No = 0	DA005_4_	h1
13	Self-rated health	0,1,2,3,4,5	r1shlt	b12
14	Health now compared with 1 year ago	worse = 1, better/same = 0	DA048	b121
15	Current smoker	No=0, Yes=1	r1smoken	d71
16	Past smoker	No=0, Yes=1	r1smokev	d72
17	Alcohol consumption	No=0, Yes=1	r1drinkr_c	d81
18	Alcohol consumption in the past	No=0, Yes=1	r1drinkev	d82
19	Falling history	No=0, Yes=1	DA023	g4c1_18
20	Hospitalization	Yes = 1, No = 0	r1hosp1y	g131
21	Activity of daily living	0,1,2,3,4,5,6		
22	Instrumental activity of daily living	0,1,2,3,4,5,6		
23	Socialization	Yes = 0, No = 1	exercise(r1ltact_c/r1mdact_c/r1vgact_c)	d91
24	Comorbidities	continuous variables	r1socwk, r1hibpe,	d11h, g14a1=1,

			1diabe, r1lunge, r1livere, r1hearte, r1stroke, r1kidneye, r1digeste, r1memrye, r1arthre, r1asthmae	g14a1=2, g14a1=6, g14a1=24, g14a1=3, g14a1=4, g14a1=19, g14a1=11, g14a1=12/g14a1=15, g14a1=14, g14a1=5
**Psychosocial Factors**				
25	Depression (CESD-10)	continuous variables		
26	Cognitive impairment	continuous variables		
27	Sleep problem	Yes = 1, No = 0	r1sleeprl	g01
28	Self-perceived loneliness	Yes = 1, No = 0	r1flonel	b24
29	Self-perceived fearful/anxious	Yes = 1, No = 0	r1fearll	b23
**Blood Related Factors**				
30	White blood cell	in thousands	qc1_vb002	wbc
31	Hemoglobin	g/dL	qc1_vb004	hgb
32	Hematocrit		qc1_vb005	hct
33	Mean corpuscular volume	fL	qc1_vb006	mcv
34	Platelets	$x10^9/L$	qc1_vb009	plt
35	C-Reactive protein	mg/L	newcrp	crphs
36	Total cholesterol	mg/dL	newcho	cho
37	Triglycerides	mg/dL	newtg	tg
38	Glucose	mg/dL	newglu	glu
39	Uric acid	mg/dL	newua	ua

Supplementary Table S3

Model performance in the internal and external validation cohort.

	CHARLS					CLHLS-HF				
**Model**	**Accuracy**	**Precision**	**Recall**	**F1**	**AUC**	**Accuracy**	**Precision**	**Recall**	**F1**	**AUC**
LR	0.576	0.491	0.767	0.599	0.634	0.627	0.548	0.535	0.541	0.675
K-NN	0.588	0.500	0.628	0.557	0.639	0.591	0.503	0.523	0.513	0.626
SVM	0.647	0.739	0.222	0.341	0.700	0.644	0.695	0.238	0.355	0.671
RF	0.658	0.554	0.866	0.676	0.799	0.701	0.611	0.750	0.674	0.758
GBM	0.723	0.633	0.775	0.697	0.814	0.658	0.563	0.750	0.643	0.758
XGBoost	0.733	0.644	0.782	0.707	0.810	0.715	0.648	0.674	0.661	0.786
CatBoost	0.738	0.630	0.876	0.733	0.854	0.687	0.594	0.756	0.665	0.772

Supplementary Table S4

TRIPOD checklist.

TRIPOD Checklist: Prediction Model Development and Validation

Section/Topic	Item		Checklist Item	Page
**Title and abstract**				
Title	1	D;V	Identify the study as developing and/or validating a multivariable prediction model, the target population, and the outcome to be predicted.	1
Abstract	2	D;V	Provide a summary of objectives, study design, setting, participants, sample size, predictors, outcome, statistical analysis, results, and conclusions.	1
**Introduction**				
Background and objectives	3a	D;V	Explain the medical context (including whether diagnostic or prognostic) and rationale for developing or validating the multivariable prediction model, including references to existing models.	1-2
	3b	D;V	Specify the objectives, including whether the study describes the development or validation of the model or both.	2
**Methods**				
Source of data	4a	D;V	Describe the study design or source of data (e.g., randomized trial, cohort, or registry data), separately for the development and validation data sets, if applicable.	2-3
	4b	D;V	Specify the key study dates, including start of accrual; end of accrual; and, if applicable, end of follow-up.	2-3
Participants	5a	D;V	Specify key elements of the study setting (e.g., primary care, secondary care, general population) including number and location of centres.	2-3
	5b	D;V	Describe eligibility criteria for participants.	2-3
	5c	D;V	Give details of treatments received, if relevant.	NA
Outcome	6a	D;V	Clearly define the outcome that is predicted by the prediction model, including how and when assessed.	3
	6b	D;V	Report any actions to blind assessment of the outcome to be predicted.	NA
Predictors	7a	D;V	Clearly define all predictors used in developing or validating the multivariable prediction model, including how and when they were measured.	3
	7b	D;V	Report any actions to blind assessment of predictors for the outcome and other predictors.	NA
Sample size	8	D;V	Explain how the study size was arrived at.	4
Missing data	9	D;V	Describe how missing data were handled (e.g., complete-case analysis, single imputation, multiple imputation) with details of any imputation method.	3
Statistical analysis methods	10a	D	Describe how predictors were handled in the analyses.	3
	10b	D	Specify type of model, all model-building procedures (including any predictor selection), and method for internal validation.	3-4
	10c	V	For validation, describe how the predictions were calculated.	4
	10d	D;V	Specify all measures used to assess model performance and, if relevant, to compare multiple models.	4
	10e	V	Describe any model updating (e.g., recalibration) arising from the validation, if done.	NA
Risk groups	11	D;V	Provide details on how risk groups were created, if done.	NA
Development vs. validation	12	V	For validation, identify any differences from the development data in setting, eligibility criteria, outcome, and predictors.	4
**Results**				
Participants	13a	D;V	Describe the flow of participants through the study, including the number of participants with and without the outcome and, if applicable, a summary of the follow-up time. A diagram may be helpful.	4-5
	13b	D;V	Describe the characteristics of the participants (basic demographics, clinical features, available predictors), including the number of participants with missing data for predictors and outcome.	4-5
	13c	V	For validation, show a comparison with the development data of the distribution of important variables (demographics, predictors and outcome).	4-5
Model development	14a	D	Specify the number of participants and outcome events in each analysis.	6
	14b	D	If done, report the unadjusted association between each candidate predictor and outcome.	NA
Model specification	15a	D	Present the full prediction model to allow predictions for individuals (i.e., all regression coefficients, and model intercept or baseline survival at a given time point).	5-9
	15b	D	Explain how to the use the prediction model.	5-9
Model performance	16	D;V	Report performance measures (with CIs) for the prediction model.	6
Model-updating	17	V	If done, report the results from any model updating (i.e., model specification, model performance).	NA
**Discussion**				
Limitations	18	D;V	Discuss any limitations of the study (such as nonrepresentative sample, few events per predictor, missing data).	10
Interpretation	19a	V	For validation, discuss the results with reference to performance in the development data, and any other validation data.	9-10
	19b	D;V	Give an overall interpretation of the results, considering objectives, limitations, results from similar studies, and other relevant evidence.	9-10
Implications	20	D;V	Discuss the potential clinical use of the model and implications for future research.	10
**Other information**				
Supplementary information	21	D;V	Provide information about the availability of supplementary resources, such as study protocol, Web calculator, and data sets.	Suppl ement ary
Funding	22	D;V	Give the source of funding and the role of the funders for the present study.	Title page

*Items relevant only to the development of a prediction model are denoted by D, items relating solely to a validation of a prediction model are denoted by V, and items relating to both are denoted D;V. We recommend using the TRIPOD Checklist in conjunction with the TRIPOD Explanation and Elaboration document.
